# Anxiety in Frontline and Non-Frontline Healthcare Providers in Kelantan, Malaysia

**DOI:** 10.3390/ijerph18030861

**Published:** 2021-01-20

**Authors:** Norhayati Mohd Noor, Ruhana Che Yusof, Mohd Azman Yacob

**Affiliations:** 1 Department of Family Medicine, School of Medical Sciences, Universiti Sains Malaysia, Kubang Kerian, Kelantan 16150, Malaysia; hayatikk@usm.my; 2 Administrative Department, Raja Perempuan Zainab II Hospital, Kota Bharu, Kelantan 15586, Malaysia; drmohdazman@moh.gov.my

**Keywords:** COVID-19, anxiety, healthcare providers, pandemic, psychological

## Abstract

In response to the coronavirus disease 2019 (COVID-19) pandemic, healthcare providers are exposed to psychological and mental health implications, including vicarious traumatization, anxiety, and depression. Gradual increases in the number of COVID-19 cases meant they were inadequately protected from contamination due to a shortage of protective equipment, excessive workloads, emotional exhaustion and frustration. These circumstances affect their work performance in delivering health services. This study aims to compare the levels of anxiety in frontline and non-frontline healthcare providers during the COVID-19 pandemic. This study applied a comparative cross-sectional design between May and July 2020 at the Hospital Raja Perempuan Zainab II. Convenient sampling was applied in the selection of eligible participants. The case report form contained two self-administered questionnaires, namely, The Hospital Anxiety and Depression Scale and Medical Outcome Study Social Support Survey. Descriptive analysis, analysis of variance, and analysis of covariance were conducted using SPSS version 26. The number of participants recruited was 306, including 160 healthcare providers in the frontline group and 146 in the non-frontline group. The non-frontline healthcare providers reported a significantly higher anxiety mean score of 1.7 than the frontline providers after adjusting for gender, duration of employment, and social support. It indicates that non-frontline healthcare providers require psychological support similar to that of frontline healthcare providers during the COVID-19 pandemic.

## 1. Introduction

On 11 March 2020, the World Health Organization (WHO) declared coronavirus disease 2019 (COVID-19) a global pandemic as a result of its immense spread worldwide [1,2]. The first cases of COVID-19 were reported as beginning in mid-November 2019 in Wuhan, China [3,4]. The first COVID-19 case in Malaysia was reported on 25 January 2020 [5]. On 18 March 2020, the Prime Minister of Malaysia announced a movement-control order after a spike in COVID-19 cases [6,7].

The COVID-19 pandemic has hugely affected frontline healthcare providers, both physically and emotionally. For example, they are highly vulnerable to experiencing physical, mental, and emotional effects, including exhaustion due to overwork, frustration, discrimination, isolation, negative patient behaviors, and lack of contact with their own families [8,9]. In addition to increased workloads due to increased numbers of patients and staff shortages, frontline healthcare providers now face the possibility of being exposed to and infected with COVID-19 [10]. The mental stress that they experience has increased significantly because they must work in relatively confined spaces, wear bulky protective clothing, and care for a large number of anxious patients [11]. This situation has been noted as causing mental health problems, including stress, anxiety, depression, insomnia, denial, anger, and fear [8,9,12,13].

In China, a study of 606 frontline hospital staff and 1099 members of the general public in 133 cities found that the rates of psychological disturbances, including depression (57.6%), anxiety (45.4%), insomnia (32.0%), and somatization symptoms (12.0%), were higher in frontline medical staff than in the general public [14]. Another study reported that approximately one-third of healthcare providers in India, especially females, those of younger ages, and those who had comorbid physical illnesses, experienced significant psychological effects [15]. In Singapore, a comparison of medical versus non-medical healthcare workers found that the rates of anxiety and stress were higher among non-medical healthcare workers than among medical ones. The reasons for this may include having less access to formal psychological support, less first-hand medical information about the pandemic, and less-intensive training on the use of personal protective equipment (PPE) and infection-control measures [16].

Anxiety is one of the psychological impacts defined as a psychiatric disorder characterized by clinically significant excesses of worry and anxiety [17]. Healthcare providers may feel anxious about having or contracting COVID-19. The model of COVID-19 health anxiety states that individuals are more likely to withdraw from the situation by suppressing those feelings that could have detrimental effects on psychological needs or impair their ability to function effectively or engage with family and experience improved well-being [18]. This theoretical model was predicted from a combination of transactional stress theory [19] and self-determination theory [20].

COVID-19 anxiety may be related to the shortages of PPE and other essential equipment that occurred in the pandemic’s early phases. It may also be related to workers’ challenges regarding childcare and other family needs as they work increased hours. Healthcare providers may experience irregular hours and higher workloads due to increased numbers of patients. This situation may worsen if some providers are deployed to new or unfamiliar clinics due to healthcare-staffing shortages. They may also face emotional strain and physical exhaustion from caring for an increased number of patients, be exposed to the critical illness or death of their patients or colleagues, and face moral dilemmas when making decisions about providing care with limited resources [21]. Other sources of anxiety include lack of access to up-to-date information and communication, worry about exposure to COVID-19 at work and taking it home to family members. There was also uncertainty about whether their organization will support them and meet their personal and family needs if they become ill [22].

Healthcare providers’ anxiety levels have been significantly associated with their stress levels, which negatively affects self-efficacy and sleep quality [23]. This type of mental health problem not only affects frontline healthcare providers’ abilities to focus, comprehend, and make decisions, which can hinder the fight against COVID-19, it may also have long-term effects on their overall wellbeing [8]. In addition, anxiety can decrease healthcare providers’ confidence in themselves and the healthcare delivery system just when their abilities to maintain calmness and reassure the public are needed the most [22].

In the fight against the COVID-19 pandemic, frontline healthcare providers who work in hospitals that directly diagnose, treat, and care for patients have faced enormous pressures, including a high risk of infection and inadequate protections from contamination, overwork, frustration, and exhaustion. These pressures impose significant psychological and mental health concerns for frontline healthcare providers globally. Protection from mental-health injury is essential to provide the best possible medical services to COVID-19 patients. To ensure healthcare providers’ preparedness during the COVID-19 crisis, it is crucial that there be a stable psychological state among frontline providers. Therefore, early professional assessment and intervention are necessary. In Malaysia, research on frontline healthcare providers’ psychological wellbeing is still in its infancy.

To the best of the authors’ knowledge, there is a minimal number of published studies involving healthcare providers’ mental health in Malaysia and notably few studies on anxiety. A study in Paris [24] extended a psychological support system to non-frontline healthcare providers after the researchers realised that non-frontline healthcare providers were also experiencing psychological distress. A few studies reported that frontline healthcare providers experienced more anxiety than non-frontline healthcare providers [25,26] and a study in Singapore [16] reported otherwise. We hypothesized that there would be a difference in the anxiety levels between frontline and non-frontline healthcare providers in Kelantan, Malaysia. Therefore, this study aimed to compare the levels of anxiety of frontline and non-frontline healthcare providers during the COVID-19 pandemic in Kelantan and was assessed using the Malay version of the Hospital Anxiety and Depression Scale (HADS) [27]. We have applied these standard and widely used scales for objective assessment of anxiety and social support. Frontline healthcare providers were those engaged in providing care for COVID-19 patients who had been identified at the healthcare facility involved.

## 2. Materials and Methods

This was a comparative cross-sectional design study conducted among healthcare providers in two hospitals managing COVID-19 cases in Kelantan, Malaysia between May and July 2020. The participants were healthcare providers, namely doctors, nurses, and medical assistants (Human Research Ethics Committee USM: USM/JEPeM/COVID19-10; Medical Research & Ethics Committee MOH: NMRR-20-703-54576 (IIR)). Both frontline and non-frontline healthcare providers were identified and invited to participate in the study. Convenient sampling was applied in the selection of eligible participants. Those diagnosed as having any psychiatric illnesses were excluded.

The sample size was obtained by comparing two means using the software Power and Sample Size Calculation version 3.0.43 (Microsoft Corp., Redmond, DC, USA, 2012). The standard deviation of the anxiety scores among the non-frontline healthcare providers was 10.6 based on a previous study [28]. The detectable difference of 3.5 was determined after considering its clinical importance. After considering the alpha of 0.05, the power of 80%, and the nonresponse rate of 10%, the calculated sample size was 160 for frontline and 160 for non-frontline healthcare providers.

The case-report form was divided into several parts, including socioeconomic data, the HADS, and the Medical Outcome Study (MOS) Social Support Survey. The sociodemographic data included age, race, marital status, number of children, education level, household income, and occupational data (occupation, duration of employment, duration of employment, shift work, and type of work).

Hospital Anxiety and Depression Scale. The HADS questionnaire is a 14-item scale that has two subscales: the Hamilton Anxiety Depression Scale-Anxiety (HADS-A) and the Hamilton Anxiety Depression Scale-Depression (HADS-D). This instrument includes 14 statements, each of which has four choices. The odd items measure anxiety level, and the even items measure depression level. The four response choices are never (0), mild (1), moderate (2), and severe (3). The possible scores range from 0 to 21. Higher scores denote increased symptoms of anxiety and depression [29]. The Malay version of the HADS was used by permission. It has been reported to have a sensitivity of 90.0% and a specificity of 86.2% for anxiety [27]. The best cut-off point is 8, meaning that anxiety is indicated if the scores for the items are 8 or above.

Medical Outcome Study Social Support Survey. The Malay version of the MOS Social Support Survey consists of four dimensions and 16 items: emotional/informational support (EMI), tangible support (TAN), affectionate support (AFF), and positive social interaction (POS) [30]. The EMI is the extent to which interpersonal relationships provide guidance, positive effect, and empathetic understanding (six items). The TAN is the provision of material aid or behavioral assistance (three items). The AFF is the expression of love and affection (three items). The POS is the availability of someone with whom to have fun (four items). Items are rated on a 5-point scale ranging from 1 (none of the time) to 5 (all the time), with higher scores indicating more support. The raw scores for each dimension and the overall functional social support scores were transformed to percent score. The composite reliability of the domains ranged from 0.649 to 0.903, the average variance extracted from the domains ranged from 0.390 to 0.699, and the Cronbach’s alpha of the domains ranged from 0.616 to 0.902. The social support was included in the analysis because it is an important confounder that may influence anxiety levels in healthcare providers.

The participants were invited using an online method: The WhatsApp application was used to distribute a Google form. Those who agreed to participate were requested to sign a virtual consent form and then complete the self-administered questionnaire. Their participation in the study was informed and voluntary, and they were permitted to withdraw at any time. They were not required to sign-in to a Google account to take the survey. Those who were identified as at risk of developing significant psychological conditions were referred to the hospital’s Psychological First Aid team for counseling and psychological support.

The data entry and analysis were conducted using IBM SPSS Statistics version 26.0 (SPSS Inc., Chicago, IL, USA, 2019). The distributions and frequencies were examined. All continuous variables were expressed as mean and 95% CI. Frequency and percentage for categorical variables were calculated. Categories with small sample size and skewed distribution were noted. Meaningful combination of categories was done when indicated. Analysis of variance (ANOVA) was done for the fixed factor. The analysis was continued with analysis of covariance (ANCOVA). The dependent variable was the anxiety scores using the frontline and non-frontline healthcare providers as a grouping variable. In the analysis, gender, duration of employment, and social support were controlled for. Since these were confirmatory analyses, the variables in the research questions and all the potential confounders were added to the model. Interactions were checked one by one with the fixed factor. Then, the ‘Preliminary final model’ was obtained.

Model assessment was done by checking the linearity assumptions (overall model linearity and linearity of each independent numerical variable), equal variance assumption, normality assumption, and outliers using standardized residual plots. Residual plots include scatter plots and histogram of residuals. In the scatter plot (residuals versus predicted), if the standardized residuals were randomly scattered along the zero line (predicted value), the model is considered fit and the overall linearity assumption met. If the standardized residuals were dispersed equally at any point of the predicted value, the equal variance assumption was considered satisfied. In the histogram, if the distribution of standardized residuals was normally distributed, the normality assumption was met. In the scatter plot (residuals versus independent numerical variable), if the standardized residuals were randomly scattered along the zero line, the variable functional form was appropriate. The adjusted means of the outcome variable were then obtained with the final model.

## 3. Results

The study used 306 participants: the frontline healthcare provider group (*n* = 160) and the non-frontline group (*n* = 146) with a response rate of 100.0% and 91.3%, respectively. The socioeconomic characteristics of the frontline and non-frontline healthcare providers are shown in Table 1.

The HADS-A items showed that the mean score of the non-frontline group was higher than that of the frontline group in most scale items. The difference between the two groups was not statistically significant except for items 9 and 13 (Table 2).

The HADS-A mean score for frontline healthcare providers was normally distributed, ranging from 1 to 16 and having a mean and standard deviation (SD) of 5.6 (2.98). The mean score for non-frontline healthcare providers was also normally distributed, ranging from 1 to 14 and having a mean (SD) of 6.9 (3.25). In accordance with our research assumption, the ANOVA showed a significant difference in anxiety scores, which were higher in non-frontline healthcare providers (*p* < 0.001) than in frontline healthcare providers (Table 3). The ANCOVA showed a significant difference between the two groups in the estimated marginal mean of the anxiety scores, with a mean difference of −1.7 after adjusting for gender, duration of employment, and social support (*p* < 0.001).

No significant interaction existed between the groups and the confounders. All model assumptions were met. The residual plots indicated that the assumptions regarding overall model fitness and equal variance were satisfied. The normality of the standardized residuals was appropriate. The variable functional form for the MOS social support score was appropriate. There was no obvious outlier when plotting standardized residuals against the predicted value. The ANCOVA analysis confirmed the higher anxiety scores of non-frontline healthcare providers after adjusting for gender, duration of employment, and social support.

## 4. Discussion

Of the COVID-19, MERS (Middle East respiratory syndrome–related coronavirus), and SARS (Severe Acute Respiratory Syndrome) infectious disease outbreaks, COVID-19 is the most severe. The current COVID-19 pandemic is ongoing, and it is not yet known how long it will last. Some countries, including Malaysia, have faced second or third waves of the infection. It has become worse since the identification of a super-spreader virus, the transmissive ability of which is overwhelming [31]. COVID-19 has heavily burdened healthcare systems globally, and the WHO has emphasized the extremely high burden placed on healthcare providers who must respond to the outbreak and has addressed the immediate need to prevent serious effects on their physical and psychological health [32].

Healthcare providers must face this challenging pandemic, which is a new and emerging phenomenon that affects their physical and psychological health. The most common psychological effects of the COVID-19 pandemic among healthcare providers are psychological distress, depression, anxiety, insomnia, and somatization symptoms [14,16,33]. This study, which aimed at anxiety specifically, found that non-frontline healthcare providers have higher anxiety levels than frontline healthcare providers, as measured using the HADS-A. This result was similar to those of a study in Singapore [16] that used the Depression, Anxiety and Stress Scale (DASS-21). However, a study in China that used the Beck Anxiety Inventory reported higher anxiety levels in frontline medical workers than in non-frontline ones [26]. Similarly, a study in Oman that used the DASS-21 reported higher anxiety levels in frontline than non-frontline healthcare workers [25]. The different outcomes of these studies may be partially accounted for by the countries’ policies and differences in the tools used to conduct the assessments [34].

Knowledge of the novel COVID-19 virus plays a role in controlling healthcare workers’ anxiety. Many completed studies and some still ongoing have gathered data on this virus worldwide. Healthcare providers need to be knowledgeable about COVID-19 and alerted to up-to-date information. Such information is distributed using a variety of media, including radio and television, social media, the websites of pertinent government authorities, and seminars and workshops [35]. New knowledge and information determine the actions needed to prepare healthcare workers to protect the healthcare system for themselves and their societies. However, non-frontline healthcare providers claim to have less access to first-hand medical information on the outbreak [16].

Knowledge also directly affects healthcare providers’ attitudes. The more knowledge they have, the more confident they can fight the pandemic [36]. The same study also found that non-frontline healthcare providers were less confident about defeating the virus than frontline healthcare providers [36]. Studies in Northwest Ethiopia [37], Vietnam [35], and Iran [38] found that healthcare providers who had good knowledge of COVID-19 had a more positive attitude than those who had poor knowledge of the infection. Good knowledge and a positive attitude combined with good practices regarding COVID-19 infection at work may help to manage workers’ anxiety levels and prevent psychological implications among healthcare providers.

During a health crisis, information from social media may be inaccurate or misleading. Most news about COVID-19 is distressing and has sometimes been associated with rumors [39]. It can trigger a community’s anxiety and heighten stress responses, resulting in psychological distress, including in non-frontline healthcare providers who lack access to first-hand medical information on the outbreak [16].

In addition, some healthcare organizations may need to deploy staff to new areas, either as frontliners or to cover shortages of healthcare staff in other roles [40]. Changes in their usual workplaces or routines may trigger anxieties in non-frontliners about their abilities to perform their new short-term responsibilities [22]. To do so, they will need to adapt to and cope with a new working environment using new knowledge and skills. If they cannot meet expectations, they will experience stress responses, which cause changes in physiology, psychology, and behavior [41], including anxiety levels. Compared to frontline healthcare providers, non-frontliners have not been well-prepared with the knowledge, skills, and psychology to face the pandemic [42].

High levels of exposure to COVID-19 infection can cause increased anxiety levels [43]. Healthcare facilities are “red zones” for COVID-19 infection [31]. Even non-frontliners are subject to nosocomial transmission, and worry about bringing the infection to their families, particularly because non-frontline healthcare providers receive less-intensive training regarding PPE and infection-control measures [16]. Adding to non-frontline healthcare providers’ anxiety levels is their increased work hours due to staff shortages and their uncertainties about childcare. They work more hours during school closures and support their families if they develop the infection and quarantine [22].

The public’s stress responses can also increase help-seeking behaviors that may overburden healthcare facilities, including panic buying of essential PPE such as face masks and hand sanitizer, which has led to global shortages of these necessities [44]. Scarcity of the PPE that healthcare providers need to perform routine care only increases their anxiety levels [22]. Frontline healthcare providers should have priority for PPE to enable a safer environment for them and their patients [45] and to prevent nosocomial infection [46].

Stigmatization and discrimination play roles in the pandemic’s psychological effects on non-frontline healthcare providers. Although they are not handling COVID-19 patients, they still may be avoided in their communities as being in “close contact” with COVID-19 patients [47], just as frontline healthcare providers are. However, frontline healthcare providers’ experiences and skills reduce their vulnerability to both COVID-19 and the stress of dealing with it [48] compared to non-frontline healthcare providers, who are less experienced in handling the pandemic. In India, there have been more than 200 reported incidents of attacks on healthcare workers and healthcare facilities related to COVID-19. That study also noted that in Malawi and Mexico, healthcare workers reported having been physically assaulted and denied access to public transportation [49].

The differences between the results of the current study and those of others may be influenced by differences in government policies, healthcare systems, and communities. Government policies and healthcare systems play roles in the early implementation and support of care that can prevent adverse psychological effects. Supportive care systems—whether psychological support such as counseling, therapy, and support systems among colleagues [16] or the provision of adequate staff and PPE—may protect from psychological effects. Non-frontline healthcare workers who had reduced access to formal psychological support reported higher mean anxiety scores [16], and heavy workloads may also trigger anxiety due to the number of cases [34,50] relative to the number of staff.

This study is not without limitations. A cross-sectional design study only concerned comparing anxiety levels between frontline and non-frontline healthcare providers and unable to determine the causal relationship between healthcare providers’ anxiety. The self-administered questionnaire, which was applied in this study may produce common methodological bias and social desirability bias. However, the anonymous survey was used with the hope it may reduce such biases.

## 5. Conclusions

Anxiety is one of the most common psychological effects during the COVID-19 public health crisis, and it is pervasive among healthcare providers. This study has proved that both frontline and non-frontline healthcare providers experienced anxiety. Non-frontline healthcare providers had higher anxiety levels compared to frontline healthcare providers. Whether healthcare providers are frontline or non-frontline, their psychological health should be protected by the provision of psychological support and improved mental-health support and care services globally. All healthcare providers play important roles in providing community healthcare during the fight against the COVID-19 pandemic.

## Figures and Tables

**Table 1:** Socio-economic characteristics of healthcare providers.

	Frontline		Non-Frontline		Chi^2^ (*df* ^c^)	*p*-Value
Variables	*n*	(%)	*n*	(%)		
Age (years) ^a^	38.0	(6.21)	38.5	(7.13)	−0.7 (304)	0.511
Household income (RM) ^a,b^	5754.7	(3121.83)	4369.1	(2726.48)	3.4 (304)	0.001
Number of children ^a^	2.5	(1.53)	2.3	(1.57)	1.0 (304)	0.305
Duration of employment (years) ^a^	12.7	(5.84)	12.4	(7.01)	0.3 (304)	0.754
Sex						
Male	19	(11.9)	42	(28.8)	13.7 (1)	<0.005
Female	141	(88.1)	104	(71.2)		
Race						
Malay	156	(97.5)	145	(99.3)	1.6 (1)	0.374 ^d^
Non-Malay	4	(2.5)	1	(0.7)		
Education level						
Diploma	134	(83.8)	135	(92.4)	6.0 (2)	0.055 ^d^
Bachelor	18	(11.2)	9	(6.2)		
Master	8	(5.0)	2	(1.4)		
Marital status						
Married	133	(83.1)	132	(90.4)	3.5 (1)	0.062
Unmarried	27	(16.9)	14	(9.6)		
Occupation						
Paramedics	137	(85.6)	139	(95.2)	7.9 (1)	0.005
Medical staffs	23	(14.4)	7	(4.8)		
Department						
Medical	54	(33.8)	24	(16.4)	154.0 (5)	<0.005 ^d^
Emergency	2	(1.3)	51	(34.9)		
Intensive Care Unit	67	(41.9)	0	(0)		
Surgical	14	(8.8)	56	(38.4)		
Obstetric Gynaecology	5	(3.1)	0	(0)		
Others	18	(11.2)	15	(10.2)		
Shift work						
No	154	(96.2)	137	(93.8)	3.9 (1)	0.048
Yes	6	(3.8)	9	(6.2)		
Social support						
Emotional support ^a^	70.3	(25.44)	58.5	(27.93)	3.9 (304)	<0.005
Tangible support ^a^	64.4	(28.21)	56.2	(29.13)	2.5 (304)	0.013
Affectionate support ^a^	74.7	(25.55)	62.8	(28.70)	3.8 (291.51)	<0.005
Positive social interaction ^a^	74.2	(24.48)	62.5	(27.51)	3.9 (291.50)	<0.005

^a^ Expressed as mean (standard deviation); ^b^ Missing values (Frontline, *n* = 128; non-frontline, *n* = 87); ^c^ degree of freedom; ^d^ Fisher exact test.

**Table 2:** Healthcare providers’ scores on the Hamilton Anxiety Depression Scale–Anxiety.

		Frontliners (*n* = 160)		Non-Frontliners (*n* = 146)		t (*df* ^b^)	*p*-Value
No.	Items	Mean	(SD) ^a^	Mean	(SD) ^a^		
1	I feel tense or “wound up.”	1.43	(0.86)	1.36	(0.98)	0.59 (304)	0.555
3	I get a sort of frightened, feeling as if something awful is about to happen.	0.96	(0.74)	1.1	(0.83)	−1.55 (304)	0.121
5	Worrying thoughts go through my mind.	0.39	(0.82)	0.94	(1.11)	−4.91 (264.76)	<0.005
7	I can sit at ease and feel relaxed.	0.93	(0.84)	0.96	(0.89)	−0.34 (304)	0.732
9	I get a sort of frightened feeling, like “butterflies” in my stomach.	0.72	(0.55)	0.93	(0.85)	−2.57 (244.41)	0.011
11	I feel restless, as if I have to be on the move.	0.74	(0.77)	0.78	(0.78)	−0.49 (304)	0.625
13	I get sudden feelings of panic.	0.46	(0.65)	0.84	(0.84)	−4.43 (304)	<0.005

^a^ Standard deviation; ^b^ degree of freedom.

**Table 3:** Comparison of frontline and non-frontline healthcare providers’ anxiety levels.

Groups	*n*	Desc Mean ^a^	(SD) ^b^	EMM ^c^ (95%CI ^d^)	Mean Diff ^e^ (95%CI ^d^)	*F* Stat ^f^ (*df* ^g^)	*p-*Value ^h^
Frontliners	160	5.6	(2.98)	4.9 (4.32; 5.48)	−1.7 (−2.39; −0.97)	21.62 (1)	<0.001
Non-frontliners	146	6.9	(3.25)	6.6 (6.05; 7.12)			

^a^ Descriptive mean. ^b^ Standard deviation; ^c^ Estimated marginal mean; ^d^ Confidence interval; ^e^ Mean difference; ^f^ *F* statistic; ^g^ Degree of freedom; ^h^ Analysis of covariance, adjusted for gender, duration of employment, and social support.

## Data Availability

All relevant data are within the manuscript.
